# Observations on Malaria

**Published:** 1884-04

**Authors:** 


### OBSERVATIONS ON MALARIA.

TO a scientific contemporary Dr. Harvey of Orange, N. J., contributes some interesting opinions on malarial diseases, which he has studied as they appear around him, in New Jersey, recording his observations with an intelligence, and with a freedom from prejudice which must make them doubly valuable. Considerable portions of the towns of Orange, Bloomfield, and Belleville lie near the banks of a small stream which is interrupted at intervals by mill dams. Of the families living in the valley of this stream, ninety per cent., according to Dr. Harvey's estimate, have been afflicted with intermittent fever at some time within the last five years. As a partial compensation for this, it is found that typhoid fever, consumption, and the eruptive fevers, are less common in the malarial district than in the healthier regions, but the constant prevalence of a disease which attacks nearly all the inhabitants at short intervals, and keeps even those who escape acute symptoms under a depressing influence, is a serious calamity.

Local opinion attributes the miasma of the valley to mill ponds, and although the mill owners strenuously deny that their ponds affect the health of the district, it is observed that in other places near by, where the water courses, though lying in a similar relation to the region about them, have not been interrupted, ague is unknown. Like a really scientific observer, Dr. Harvey has noted all the circumstances, whether important or unimportant in appearance, attending the presence of persistent malarial poisoning, and has become convinced, notwithstanding the prejudice to the contrary, which he at first entertained, that the character of the drinking water used, has something to do with the communication of the fever.

Nearly all the households in the infected district obtain water from shallow wells sunk a few feet into the saturated ground—and suffer from chills. One proprietor, however, about three years ago built a cistern for rain water, and has since employed the cistern water entirely for domestic use. During that time his family has been entirely exempt from fever, but before the well was abandoned there was always one or more cases in the house. Another family which had for years used a shallow well, and suffered from continual chills, about eighteen months ago had a pipe well driven to a depth of about sixty feet, penetrating the sandstone which underlies the region. Since the new well came into use malaria has forsaken the house.

